# Resilience and hope during advanced disease: a pilot study with metastatic colorectal cancer patients

**DOI:** 10.1186/s12904-016-0139-y

**Published:** 2016-08-02

**Authors:** Joao Paulo Consentino Solano, Amanda Gomes da Silva, Ivan Agurtov Soares, Hazem Adel Ashmawi, Joaquim Edson Vieira

**Affiliations:** 1 Hospital das Clínicas of Universidade de São Paulo Medical School, Av. Dr. Enéas de Carvalho Aguiar, 44 (INCOR), 2° andar, bloco I, Zipcode: 05403-900 São Paulo, Brazil; 2 A.C. Camargo Cancer Center, São Paulo, Brazil; 3 Centro Universitário São Camilo Medical School, São Paulo, Brazil; 4 Department of Anesthesiology, Universidade de São Paulo Medical School, São Paulo, Brazil

**Keywords:** Cancer, Oncology, Resilience, Psychological, Hope, Terminal care, Palliative care

## Abstract

**Background:**

The balance between hope-hopelessness plays an important role in the way terminally ill patients report quality of life, and personal resilience may be related to hope at the end of life. The objective of this study was to explore associations between personal resilience, hope, and other possible predictors of hope in advanced cancer patients.

**Methods:**

A cross-sectional pilot study was carried out with metastatic colorectal cancer patients in a tertiary hospital. The patients answered the Connor-Davidson Resilience Scale, Herth Hope Index, Barthel Index, an instrument addressing family and social support, visual-numeric scales for pain and suffering, a two-item screening for depression, socio-demographic and socio-economic information about the family.

**Results:**

Forty-four patients were interviewed (mean age 56 years; range 29-86). A strong correlation was noted between resilience and hope (0.63; *p* < 0.05). No correlation was found between hope and independence for activities of daily living, support from family and community, and pain and suffering levels. Of the 44 patients, 20 presented with depressive symptoms. These depressive patients had lower resilience (*p* = 0.005) and hope (*p* = 0.003), and higher scores of suffering (*p* < 0.001). The association between resilience and hope kept stable after adjusting for age, gender, and presence of depression (*p* < 0.001).

**Conclusion:**

Given that resilience is a dynamic, changeable path that can improve hope, resilience-fostering interventions should be most valued in palliative care settings and should be commenced as soon as possible with cancer patients. Patients with advanced stages of non-malignant conditions would also probably benefit from such interventions.

**Electronic supplementary material:**

The online version of this article (doi:10.1186/s12904-016-0139-y) contains supplementary material, which is available to authorized users.

## Background

In oncology and palliative care, the balance between hope-hopelessness may play an important role in the way patients report quality of life and quality of dying [1–3]. Hopelessness and other features of spiritual distress may be mismatched with depression, leaving a considerable number of patients tackling existential concerns and discomforts by themselves, despite receiving antidepressants on a regular basis [4, 5].

Spiritual distress is a highly valued concept in nursing care. It is defined by the North American Nursing Diagnosis Association as a state of “disruption in the life principle that pervades a person’s entire being and that integrates and transcends his or her biological and psychological nature” [6] (p.67). In our context, Caldeira and colleagues validated the diagnosis of spiritual distress in a sample of forty-five elderly cancer patients; in that study, the prevalence of spiritual distress was as high as 42 % [7]. Hopelessness is regarded as a sign indicative of spiritual distress [8, 9]. Anandarajah and Hight [10] note that spiritual distress occurs when a person is “unable to find sources of meaning, hope, love, peace, comfort, strength, and connection in life or when conflict occurs between their beliefs and what is happening in their life”(pp. 83-84). Morita, in a study with hospice patients, identified three factors in connection with existential/spiritual distress: loss of autonomy, lowered self-esteem, and hopelessness [11]. Hopelessness, as a source of spiritual distress, may seriously contribute to deteriorate the wellbeing of terminally ill cancer patients [12]. Chochinov found in a sample of 196 terminal cancer patients that hopelessness was more highly correlated with suicidal ideation than was depression [13]. Among dying patients, hope is apparently related to concepts of meaning and purpose [14]. Duggleby [15] reported that maintaining hope was a way for terminally ill patients to endure and cope with their suffering. Hopelessness as an experience among palliative care patients has the power to undermine a sense that life has ongoing value or intrinsic worth [12].

Since the early 1990’s, Herth has developed assessment tools to measure individual hope in clinical settings. She postulates that such an instrument must apprehend the multidimensionality of the hope construct, instead of only a time-oriented, future focused dimension of hope. So, the last version of the Herth scale includes items focusing on a more global, non-time oriented sense of hope, and hope despite diminished or absent interpersonal relationships, for example [16]. This broader conceptualization of hope is more aligned with the needs of patients facing imminent death. According to clinicians and researchers in palliative care, the sense of hope in a terminally ill patient is usually (or should be) transformed, what may involve acknowledging life the way it is, searching for meaning and positive reappraisal [17]. For patients nearing death, maintaining hope is intimately connected with a sense that life continues to serve some purpose or hold meaning [14]. For these patients, hope is defined in terms of hope for no more suffering, living each day, for a peaceful death, and hope for their families [18]. Reports about comfort and well-being in terminal cancer patients have indicated that maintenance of hope as death approaches may be seen as an indicator of quality of life and quality of dying; thus, dying well seems to be connected with keeping hope until the end [1, 3, 19].

The investigation of comfort and well-being in terminal cancer has also emphasized that symptom burden and functionality constraints are negatively correlated with quality of life at the end of life [20, 21]. On the other hand, family and social support are positively correlated with quality of life at the end of life [22, 23].

Also related to spiritual wellbeing, personal resilience is a construct associated with the ability to adapt when challenged by stressors or adversities, or to strive despite the difficulty of an experienced circumstance [24, 25]. The concept of resilience is far from being a consensus among authors [26, 27]. Deshields summarizes that resilience is variably regarded as a process, a personality trait, a dynamic developmental process, an outcome post-adversity, or a combination of the above [27]. Windle, while accepting the difficulties in accurately defining resilience, proposes that it stands for “the process of negotiating, managing and adapting to significant sources of stress or trauma” [28] (p.159). Resilience was first studied in individuals who had endured significant adversity or abuse. However, in recent years, chronic stressors (e.g. ill-health and the burdens imposed by chronic life-limiting diseases) have also been a focus for the study of individual resilience [27–29].

According to theories as well as research, some features usually displayed by resilient people have been reported: hardiness, self-esteem, realistic optimism, high positive emotionality, sense of purpose in life, spirituality, moral compass, use of active coping strategies (such as problem solving and planning), ability to find meaning even in traumatic experiences, the tendency to perceive stressful events in less threatening ways and to reframe adverse experiences in a more positive light, and so forth [30–32]. Rutter proposed a model to understand resilience as a dynamic process in which personality traits interact with environmental factors to enhance or decrease resilience throughout one’s life [33]. In review papers, the majority of researchers on the topic acknowledge this model of resilience as a process [24, 29]. According to Coughlin [34], cancer survivors seem to be resilient individuals and some are likely to have an experience of subtle inner growth while facing cancer symptoms and the burdens of its treatments and limitations.

In the present manuscript, we acknowledge the model of resilience as a process, thus amenable to change over the life span. Also aligned with the work of Connor and Davidson (whose resilience scale was adapted for the context of our study), we accept that spirituality may be constitutive of a resilient self [35]. Spirituality here is taken as a broader construct in relation to religiosity [36]. Indeed, while developing a measure for resilience, Connor and Davidson included items to assess spirituality – in their words, “faith and a belief in benevolent intervention” – as a dimension of the resilience process [35] (p.77).

The association between resilience and hope is not clear in the literature. While it is common place to state that hope belongs to the domain of spirituality and spirituality is an ingredient of resilience, some researchers on resilience usually point out that hope is constitutive of a resilient self [37, 38], but others do not [31]. In the present pilot study we tested the association between resilience and hope in a sample of advanced colorectal cancer (CRC) patients. We hypothesized a positive strong association between the two constructs; we also hypothesized that resilience would be a better predictor of hope in comparison with other variables, such as intensity of pain, severity of physical limitations (functionality), and family and social support.

## Methods

### Sample size

To detect a correlation of 0.50 at 90 % power and an alpha of 0.05, we estimated that at least 38 patients should be invited to participate in the study. It was assumed that a correlation of 0.50 is moderate, according to Callegari-Jacques and Abramson [39, 40]. A 15 % margin was added to prevent for losses due to response inconsistencies.

### Study subjects, consent, permissions, and procedures

Between October 20, 2014 and February 2, 2015 advanced CRC patients of the department of Clinical Oncology of a tertiary teaching hospital were approached. The responses of the study subjects are shown as an additional file presented with this manuscript (Additional File 1). Consecutive patients from the oncology wards were potentially eligible, unless they demonstrated unwillingness to participate. The aim was conducting complete and valid interviews with a convenience sample of 42 to 44 patients. Medical charts were checked to confirm the disease stage, sites of metastasis, and comorbid conditions. Patients with limitations to oral communication or cognitive deficits were not eligible (exclusion criteria of the study protocol). Detection of cognitive deficits was done by means of the Confusion Assessment Method (CAM), a widely used assessment tool to screen for cognitive deficits. For the present study, a Brazilian Portuguese adapted and validated CAM version was used [41]. If the CAM was positive, the interview was interrupted delicately.

All participants consented to become subjects of the study and answered the questionnaires by themselves in the presence of the interviewer who previously had read each study item and response options to the patient (assisted application). At the beginning of the interview, patients were asked about socio-demographic characteristics, including ability to read and number of years’ formal schooling. At the end, they were asked about socio-economics of their family, according to an A to E Brazilian strata classification, A being wealthier families [42].

### Measures

#### Perceived support from family and community

For the purpose of the present study, a chart recorded information regarding the household constitution (number of people the patient lived with, their names, and category of proximity), and other recorded information on other support people valued by the patient, though not part of the nuclear family (again including names). For each name, the participant was invited to ascribe on a visual-numeric scale ‘how much support could be promptly available from that person’, 0 being ‘no support’ and 10 ‘all that I come to need’. Scores for each person named were then summed and the sum was divided by the number of people named in each of the charts (nuclear family and other relatives/friends). So, two mean scores were generated (varying from 0 to 10), and the variables were respectively labeled ‘family support’ and ‘community support’. (Additional File 2).

#### Connor-Davidson Resilience Scale (CD-RISC)

The full-length scale [35] was chosen, since it is currently one of the most used resilience measures worldwide. This 25-item CD-RISC varies from 0 to 100, the higher scores indicating greater resilience. A Brazilian Portuguese culturally adapted version of the CD-RISC has been factor analyzed and exhibited appropriate psychometric properties (Cronbach’s alpha 0.93; intraclass correlation coefficient 0.86) [43].

#### Herth Hope Index (HHI)

The HHI was developed in 1990 and has been extensively used to evaluate hope, particularly in palliative care clinical settings [16]. With its 12 items, the HHI varies from 12 to 48, the higher scores indicating greater hope. A Brazilian Portuguese HHI version has been available since 2008, with adequate psychometrics (Cronbach’s alpha 0.83; intraclass correlation coefficient 0.70) [44].

#### Barthel Index (BI)

The BI was proposed by Mahoney and Barthel [45] and is frequently used in oncology and gerontology to assess one’s level of dependency in relation to daily life activities. The BI has ten items and varies from 0 to 100, the higher scores indicating less dependency. Its Brazilian Portuguese version was developed by Cincura et al. [46] in a sample of post-stroke patients and tested again by Minosso et al. in a sample of elders [47].

#### Visual-numeric pain scale

Respondents scored their pain intensity (during the last 24 h) on a 0-10 scale.

#### Visual-numeric scale for experienced suffering

Respondents scored the intensity of their experienced suffering (during the last 24 h) on a 0-10 scale.

#### Two-item screening for depression

Respondents were asked “are you down or depressed?” and “are you feeling loss of interest in your activities?”, according to a proposal by Chochinov [48, 49].

### Statistical methods

Descriptive statistics established demographics and clinical characteristics of the sample. Spearman correlation coefficients were calculated for the study variables. The sample was stratified into two subgroups: one without depression and one with depression. The main study variables were compared within these subgroups using the Mann-Whitney test. A generalized linear model with gamma distribution was used to adjust the association between resilience and hope by sex, age, and presence of depression. A statistical significance level of 0.05 was adopted. Statistics were performed using the SPSS statistical package for Windows, version 17.0 [50].

## Results

Forty-four patients were interviewed. The patients had been diagnosed with colorectal cancer 3.5 years (mean) before the interview (range one month to 13 years). Mean age of the sample was 56 years (standard deviation 12.8; median 59; range 29-86). Most of the patients were married men. Fifty per cent of the sample had at least 15 years formal schooling. More than half endorsed that they could read well. No patients belonged to the deprived strata D and E, according to the Brazilian socioeconomic classification (Table 1). Table 1 also shows the encountered sites of metastases and the comorbid conditions in the sample.

**Table 1:** Socio-demographic and clinical characteristics of the sample

	Number	Percent
Gender		
Male	28	63.6
Female	16	36.4
Civil state		
Married	33	75
Single	2	4.5
Divorced	7	16
Widow/er	2	4.5
Years of formal schooling		
0-4	2	4.5
5-8	3	7
9-11	12	27.5
12-14	5	11
≥ 15	22	50
Self-rated ability to read^a^		
Can read reasonably well	4	9.5
Can read well	24	54.5
Can read very well	15	34
Socio-economic stratum		
A1	1	2
A2	14	32
B1	14	32
B2	12	27
C1	3	7
Metastases		
Liver	28	64
Lymph nodes	14	32
Peritoneum	12	27
Lung	7	16
Pelvic organs	5	11
Bone	2	5
Adrenal gland	1	2
Comorbidities		
Hypertension	11	25
Diabetes	6	14
Depression	3	7
Ischemic heart disease	2	5
Stroke	1	2
HIV+	1	2
Anxiety	1	2
Fibromyalgia	1	2
Dyslipidemia	1	2
COPD	1	2

^a^Missing information from one subject; COPD, chronic obstructive pulmonary disease

Table 2 presents the Spearman correlation coefficients among the study variables. A strong correlation appears between resilience and hope (0.63; *p* < 0.05) and a weak correlation appears between resilience and independence (0.3; *p* < 0.001).

**Table 2:** Spearman correlation coefficients among the main study variables

	Resilience	Hope	Independence	Family support	Community support	Pain
Hope	0.63 ^*^	1.00				
Independence	0.30 ^**^	0.07	1.00			
Family support	-0.01	0.06	0.11	1.00		
Community support	-0.02	0.06	-0.10	0.21	1.00	
Pain	-0.05	-0.07	-0.11	0.08	0.15	1.00
Suffering	-0.24	-0.20	0.02	0.13	0.13	0.21

^*^ *p* < 0.05

^**^ *p* < 0.001

In Table 3 the sample was stratified in patients without depression and patients with depression, the latter being less resilient (*p* = 0.005), less hopeful (*p* = 0.003), and more prone to report higher levels of suffering (*p* < 0.001).

**Table 3:** Comparison between the subsample without depression and the subsample with depression

	Median (quartiles)		Mann-Whitney test	
	No depression (*N* = 24)	Depression (*N* = 20)	Z*	p*
Years from diagnosis	3.0 (1.0-5.0)	2.0 (1.0-4.8)	- 0.663	0.51
Family support	9.5 (7.4-10.0)	9.3 (7.7-10.0)	- 0.013	0.99
Community support	9.9 (8.3-10.0)	9.5 (8.1-10.0)	- 0.274	0.78
Resilience	88.5 (74.5-93.5)	74.0 (65.0-80.0)	- **2.795**	**0.005**
Hope	43.5 (38.5-46.0)	39 (36.3-41.8)	- **3.005**	**0.003**
Independence	97.5 (85.0-100.0)	100 (61.3-100)	- 0.331	0.74
Pain	0.5 (0-3.0)	3.5 (0-7.0)	- 1.539	0.123
Suffering	2.0 (0-4.0)	6.0 (4.3-8.0)	- **4.064**	**<0.001**

* Bold data indicate statistical significance

Table 4 shows that, in the adjusted model, the association between resilience and hope is not dependent on age, sex, or having/not having depression (*p* < 0.001).

**Table 4:** Association resilience-hope adjusted for gender, age, and presence of depression

Factor	Coefficient	St error	Test’s statistics	df	p*
Intercept	23,61	4,62	26,14	1	**<0,001**
Gender (male)	0,11	1,09	0,01	1	0,924
Age (years)	0,03	0,04	0,82	1	0,365
Depression	-1,49	1,20	1,54	1	0,214
Resilience	0,20	0,05	17,95	1	**<0,001**

Generalized Linear Model with gamma distribution

*df* degrees of freedom

*p*, significance level

* Bold data indicate statistical significance

## Discussion

This pilot study with advanced CRC patients confirmed a strong association between individual resilience and hope. No association was found between independence for activities of daily living and hope, or between social support and hope. The subsample of depressed patients presented with lower levels of resilience and hope, and higher levels of reported suffering.

These findings suggest that interventions to improve hope at the end of life could be replaced by those aimed at improving personal resilience. This alternative approach might be advantageous because terminally ill individuals are prone to becoming easily bothered by common places to enhance hope (for example, “you have to keep fighting”; or “everything happens for a reason; this too shall pass”). The jargon used to communicate with severely ill people has been subdued by semantic erosion, and now offer some clichés about hope/hopefullness that are no longer useful for the patients. In this sample of advanced CRC patients, hope was related to resilience but not to other variables usually deemed important, such as level of independence, perceived support from family and community, pain and suffering (Table 2). The strong relation between hope and resilience was kept after adjusting for gender, age, and presence of depression (Table 4).

For the patient, keeping hope while facing impending death is of utmost importance. Palliative care researchers and practitioners sometimes tend to equate hope with quality of life (and quality of death) [51, 52]. If resilience is amenable to improvement, and if there is a means to intervene over one’s level of resilience, and if there is a strong association between resilience and hope, such interventions to enhance resilience should be a necessary part of treatment.

Some authors have designed interventions to enhance resilience among cancer patients. Friborg [53] developed a group psychotherapy program for breast cancer in which patients are taught better skills to alleviate stress and reinforce resilience factors. Nelson et al. [54] proposed some psychotherapeutic strategies for enhancing resilience and quality of life of adolescents with cancer (the Resilience Enhancement Adolescent Profile), music therapy being one of these strategies. Burns [55] and Robb [56] also developed a video-music intervention aimed at improving resilience and diminishing symptom distress among adolescents and young adults undergoing stem-cell transplantation.

It is also possible that other psychotherapeutic approaches that address hope at the end of life in cancer patients do so by means of strengthening resilience, although not explicitly [28]. Breitbart and colleagues [57, 58] created a program of meaning-centered group psychotherapy to advanced cancer patients that could well be an example. While addressing topics on hope and meaning with patients [59] – embedded in Viktor Frankl’s biographical accounts on survivorship from Nazi camps – it is very possible that resilience factors are being propelled in those patients. Again, derived from Frankl’s work, the meaning-making intervention [60] allegedly improves cancer patients’ self-esteem, optimism, and self-efficacy. Since these three constructs are supposed to be ingredients of personal resilience, it seems plausible to think such an approach could be viewed as resilience-fortifying. Also, the method proposed by Kissane et al. (cognitive existential group therapy) [61], whilst addressing the patient’s concepts of mastery, hope, and quality of life when coping with cancer, seems to deal with one’s resilience. The work of Chochinov in developing Dignity Therapy for advanced cancer patients has also to be cited [62]. While discussing themes such as hope, maintenance of pride, role preservation, continuity of self (and so forth) with advanced cancer patients, these patients probably become more resilient. Actually, the author listed “resilience/fighting spirit” as one of the subthemes among the dignity-conserving perspectives of the Dignity Model [12].

In a previous review, we advocated that the burdens of approximating death by a malignant condition are by no means different from approximating death by other chronic non-malignant diseases, such as aids, heart disease, chronic obstructive pulmonary disease and renal disease [63]. Therefore, one could expect approaches to enhance resilience and hope at the end of life also to be beneficial to patients dying from chronic non-malignant conditions. Indeed, Evers et al. have reviewed some interventions to reduce stress and promote resilience among patients with chronic rheumatic diseases [64]. Brown and Gerbarg [65] proposed meditation and Yoga breathing for augmenting stress resilience in geriatric samples. There is still much room to test resilience-fortifying approaches, with cancer or non-cancer patients.

The present pilot study, with its cross-sectional design, is ineffective in asserting that personal resilience predicts hope. It is possible that hope is one more ingredient of resilience. Nonetheless, other authors have indicated that resilience is predictive of hope at the end of life. Some resilience factors, like self-esteem, connectedness with significant others, and spiritual connectedness, are regarded as predictors of higher levels of hope among terminally ill cancer patients [66]. Miller [67] also suggested that higher levels of hope are anteceded by optimism, sense of meaning, and connectedness with God, which are thought to be constitutive of resilience as a construct. It is plausible to think that the above mentioned therapeutic approaches designed to help cancer patients better cope with their disease, limitations and treatment burdens, are resilience-fostering strategies that can ultimately enhance hope at the end of life.

Besides the study design, the findings presented here are also limited by the sample size. Nevertheless, this is unlikely to have influenced the strong association elicited between resilience and hope that could have been detected even in smaller samples. Going beyond this pilot study, researching larger samples of oncologic and non-oncologic patients will probably test again the association between hope at the end of life and personal resilience, as with other potential hope predictors, like social support, pain, functional status, and physical limitations. This study confirmed an initial hypothesis on the existence of an association between resilience and hope at the end of life – one that is stronger than that between social support and hope, or severity of limitations and hope. Another limitation of this study is due to the fact that the measure of social support utilized was not validated. Further studies will be necessary to test the adequacy, reliability and validity of simple visual-numeric scales to assess social support, as perceived by highly debilitated patients.

## Conclusion

This pilot study found an association between resilience and hope in a sample of advanced CRC patients. It is plausible to expect this association to be replicable in samples of patients with other cancers and also among patients facing advanced stage non-malignant conditions. There is much room for further and larger studies, in different cultural settings.

Considering an association between resilience and hope, resilience being a process amenable to change over time, resilience building interventions should be systematically tested in palliative care to evaluate certain outcomes, like hope and quality of life. After confirmed a desirable effect, such interventions could become common practices in palliative care facilities, and should commence as soon as possible, either to cancer or non-cancer patients assisted by hospice and/or palliative care teams.

## Abbreviations

BI, Barthel Index; CAM, confusion assessment method; CD-RISC, Connor-Davidson Resilience Scale; COPD, chronic obstructive pulmonary disease; CRC, colorectal cancer; HHI, Herth Hope Index

## Additional files

Additional file 1: Study data set after exclusion of participants personal identifiers. (XLSX 24 kb) Additional file 2: Charts to record "Family and Community Support" as perceived by the respondent. (DOC 53 kb)
