# Supplementary material for: Resilience and hope during advanced disease: a pilot study with metastatic colorectal cancer patients
Source: BMC Palliat Care. 2016 Aug 2;15:70. doi: 10.1186/s12904-016-0139-y (PMC4971726; doi:10.1186/s12904-016-0139-y)
Supplement: Additional file 2: — Charts to record "Family and Community Support" as perceived by the respondent. (DOC 53 kb) [file 12904_2016_139_MOESM2_ESM.doc]

**Family and Community support questionnaire**

1. How many people have you been living with? _____

1a [*Participant lives alone >>> ask:*

*How long have you been living by yourself?*]

______ (months)

2. Could you please tell me the names of those people you live with?

[*Record their names and relativeness – for example: Mary, niece*]

___________________________________________________________________________________________________________________________________________________________________________________________________________________________________________________________________________________________________________________________________________________________________

[*For each of the named persons, show the participant the visual-numeric scales below, and ask him/her to choose a scale point-number while you say the following: “How much can you rely on …(name)…to help you in any of your needs?”*]

(name)___________:

|  |  |  |  |  |  |  |  |  |  |
| --- | --- | --- | --- | --- | --- | --- | --- | --- | --- |

1 2 3 4 5 6 7 8 9 10

**little can rely on for everything**

(name)___________:

|  |  |  |  |  |  |  |  |  |  |
| --- | --- | --- | --- | --- | --- | --- | --- | --- | --- |

1 2 3 4 5 6 7 8 9 10

**little can rely on for everything**

(name)___________:

|  |  |  |  |  |  |  |  |  |  |
| --- | --- | --- | --- | --- | --- | --- | --- | --- | --- |

1 2 3 4 5 6 7 8 9 10

**little can rely on for everything**

(name)___________:

|  |  |  |  |  |  |  |  |  |  |
| --- | --- | --- | --- | --- | --- | --- | --- | --- | --- |

1 2 3 4 5 6 7 8 9 10

**little can rely on for everything**

(name)___________:

|  |  |  |  |  |  |  |  |  |  |
| --- | --- | --- | --- | --- | --- | --- | --- | --- | --- |

1 2 3 4 5 6 7 8 9 10

**little can rely on for everything**

3. Are there other relatives or friends with whom you have been in contact to? Could you tell me their names?

*[Any of the mentioned people must be named below]*

________________________________________________________________________________________________________________________________________________________________________________________________________________________________________________________________________________________________________________________________________________________________________________________________________________________________________________________________________________________________________________________

[*For each of the named persons, show the participant the visual-numeric scales below, and ask him/her to choose a scale point-number while you say the following: “How much can you rely on …(name)…to help you in any of your needs?”*]

(name)___________:

|  |  |  |  |  |  |  |  |  |  |
| --- | --- | --- | --- | --- | --- | --- | --- | --- | --- |

1 2 3 4 5 6 7 8 9 10

**little can rely on for everything**

(name)___________:

|  |  |  |  |  |  |  |  |  |  |
| --- | --- | --- | --- | --- | --- | --- | --- | --- | --- |

1 2 3 4 5 6 7 8 9 10

**little can rely on for everything**

(name)___________:

|  |  |  |  |  |  |  |  |  |  |
| --- | --- | --- | --- | --- | --- | --- | --- | --- | --- |

1 2 3 4 5 6 7 8 9 10

**little can rely on for everything**

(name)___________:

|  |  |  |  |  |  |  |  |  |  |
| --- | --- | --- | --- | --- | --- | --- | --- | --- | --- |

1 2 3 4 5 6 7 8 9 10

**little can rely on for everything**

(name)___________:

|  |  |  |  |  |  |  |  |  |  |
| --- | --- | --- | --- | --- | --- | --- | --- | --- | --- |

1 2 3 4 5 6 7 8 9 10

**little can rely on for everything**
